# Myxoedema Coma in a Medically Compliant Young Patient Presenting With Cardiogenic Shock and Requiring Cardiopulmonary Resuscitation

**DOI:** 10.7759/cureus.80140

**Published:** 2025-03-06

**Authors:** Jaskarn Virk, Ayesha Kiran, Kaksha Parrikh

**Affiliations:** 1 Internal Medicine, Robert Wood Johnson University Hospital, Rahway, USA; 2 Medicine, Government Medical College, Bhavnagar, IND

**Keywords:** altered mental status, cardiogenic shock, cardiopulmonary resuscitation, endocrine emergency, hypothyroidism, myxedema coma

## Abstract

Myxedema coma, a rare and life-threatening complication of severe hypothyroidism, manifests with altered mental status, hypothermia, bradycardia, hypotension, hyponatremia, and respiratory failure, potentially leading to multisystem organ failure. Cardiogenic shock associated with myxoedema coma, resulting from impaired myocardial contractility and reduced cardiac output, is a severe complication associated with increased mortality risk. This endocrine emergency requires prompt recognition and aggressive management. We report a case of a 30-year-old female with a history of hypothyroidism and pituitary tumor resection who presented with lethargy and experienced pulseless electrical activity cardiac arrest, with return of spontaneous circulation (ROSC) after two rounds of epinephrine. Laboratory findings revealed severe hypothyroidism, elevated thyroid-stimulating hormone (TSH), and acute kidney injury. Initial management included intravenous levothyroxine, hydrocortisone, vasopressors, and mechanical ventilation for cardiogenic shock. Echocardiography demonstrated severely reduced left ventricular ejection fraction, which gradually improved with treatment. The patient’s neurological status and thyroid function returned to normal over several days, emphasizing the importance of multidisciplinary care. This case underscores the critical role of early thyroid hormone replacement, titration of thyroid hormones, addressing precipitating factors like adrenal insufficiency and hypoglycemia, and providing intensive supportive care. Long-term management focused on medication adherence, patient education, thyroid hormone titration, and regular endocrinology follow-up to prevent complications of severe hypothyroidism. This report highlights the importance of rapid diagnosis and individualized treatment to optimize outcomes for patients with myxedema coma, a condition associated with high mortality rates despite appropriate intervention.

## Introduction

Myxedema coma is a rare, life-threatening manifestation of severe hypothyroidism, characterized by altered mental status, and hypothermia, which can lead to eventual multisystem organ failure. Patients may present with bradycardia, hypotension, hyponatremia, or hypercapnia [1,2]. Diagnosis is primarily clinical, supported by laboratory findings of severe hypothyroidism including but not limited to abnormalities in thyroid-stimulating hormone (TSH), free thyroxine (T4), and cortisol.

Management of myxedema coma includes intravenous levothyroxine, typically initiated at a dose of 200-400 mcg, followed by daily maintenance doses of 100-150 mcg. Stress-dose hydrocortisone (100 mg every 8 hours) is standard to address potential adrenal insufficiency. Mechanical ventilation, fluid resuscitation, and rewarming measures are often required for supportive care. Long-term management focuses on maintaining euthyroid status and preventing recurrence [1]. The prevalence of myxoedema coma is generally higher in older adults, particularly women with an estimated incidence of 0.22 cases per million annually [3].

Cardiogenic shock in the presence of myxedema coma is a severe complication characterized by inadequate tissue perfusion, presenting as low ejection fraction, and hypotension. This condition necessitates rapid intervention with vasopressors, inotropic agents (e.g., norepinephrine, dopamine), and aggressive supportive care to restore adequate tissue perfusion and hemodynamic stability. This condition represents a significant healthcare burden due to its high mortality rate and intensive care requirements.

This study presents a unique instance of myxedema coma in a young, medically compliant female patient in her 30s. Despite her adherence to hydrocortisone therapy, adrenal insufficiency contributed to severe hypoglycemia, which likely precipitated hypothyroidism, leading to myxoedema coma. While the TSH level was unexpectedly low, consistent with secondary hypothyroidism from prior pituitary resection, this finding complicated the diagnostic process. By exploring the pathophysiological mechanisms and clinical management of this case, we aim to contribute to the understanding of this rare but severe condition. This report emphasizes the critical role of tailored interventions, including vasopressor support, stress-dose steroids, and thyroid hormone replacement, in achieving recovery.

## Case presentation

Here, we present a case of a female in her 30s with a past medical history of hypothyroidism on Synthroid 150 mcg daily and a history of pituitary tumor resection (2014) with subsequent adrenal insufficiency managed with hydrocortisone 10 mg daily, medically compliant, who was brought in by ambulance to the emergency department (ED) for being found unconscious and having agonal breathing at home. As per EMS, the patient was found to be in severe hypoglycemia and suffered pulseless electrical activity (PEA) cardiac arrest en route with a return of spontaneous circulation (ROSC) after two rounds of epinephrine. Blood glucose was <10 mg/dL and a dextrose (D10) ampule was given en route. Prior to this episode, the family reported that the patient was in her usual state of health and had been medically compliant.

In ED, the initial vital signs were as follows: blood pressure 93/58 mmHg, heart rate 99 beats/min, SpO_2_ 91%, and respiratory rate 21 breaths/min. Glasgow Coma Scale score of 5, E3: eye response (opens eyes to verbal command), V1: verbal response (no verbal response, only incomprehensible sounds), M1: motor response (no motor response) (E3V1M1). The patient had hypothermia (core body temperature, 34.7°C). Physical examination revealed a well-kempt, age-appropriate female with periorbital edema and lethargy, yet maintaining an airway. The patient was very stuporous and would only open her eyes upon being called by her name, but did not follow commands and had a sluggish pupillary reaction.

Laboratory tests revealed significant abnormalities consistent with myxedema coma. These included hyponatremia (132 mmol/L), hypokalemia (3.1 mmol/L), and severe hypothyroidism as evidenced by a markedly reduced free triiodothyronine (free T3) level of 0.6 pg/mL and free thyroxine (free T4) of 0.17 ng/dL, with a thyroid-stimulating hormone (TSH) level of 0.158 mIU/mL.

Low TSH, free T3, and free T4 correlate with secondary hypothyroidism secondary to pituitary tumor resection. Acute kidney injury (AKI) was suggested by elevated blood urea nitrogen (BUN) of 34 mg/dL, creatinine of 2.62 mg/dL, and an estimated glomerular filtration rate (eGFR) of 24.2 mL/min/1.73 m^2^. AKI is likely due to hypoperfusion resulting from cardiogenic shock, leading to a reduction in glomerular filtration rate (GFR) and impaired sodium reabsorption, which exacerbates electrolyte imbalances, including hyponatremia.

Lactic acidosis was indicated by a lactate level of 5.4 mmol/L. Reduced cardiac output and systemic vasoconstriction, secondary to cardiogenic shock associated with myxedema coma, decreased tissue oxygenation, leading to the accumulation of lactic acid. Alternative causes like sepsis or ischemic bowel were excluded based on negative infection markers and imaging studies.

Elevated aspartate aminotransferase (AST) of 56 U/L and troponin of 435 U/L. Elevated troponin represents myocardial stress secondary to cardiogenic shock, rather than ischemia, supported by echocardiography findings of reduced ejection fraction. These findings are available in Table 1.

**Table 1:** Patient's laboratory test results day one. BUN: blood urea nitrogen; eGFR: estimated glomerular filtration rate; AST: aspartate aminotransferase; T4: thyroxine; TSH: thyroid-stimulating hormone; T3: triiodothyronine

Laboratory test	Results	Reference range
Sodium	132 mmol/L	136-145 mmol/L
Potassium	3.1 mmol/L	3.5-5.10 mmol/L
BUN	34 mg/dL	7-18 mg/dL
Creatinine	2.62 mg/dL	0.6-1.3 mg/dL
eGFR	24.2 mL/min/1.73 m^2^	>60 mL/min/1.73 m^2^
AST	56 U/L	15-37 U/L
Calcium	7.7 mg/dL	8.5-10.1 mg/dL
Lactate	5.4 mmol/L	0.4-2.0 mmol/L
Troponin	435 U/L	21-232 U/L
T4	0.17 ng/dL	0.76-1.46 ng/dL
TSH	0.158 UIU/mL	0.358-3.74 UIU/mL
Free T3	0.6 pg/mL	2.0-4.4 pg/mL

Arterial blood gas (ABG) findings were added in Table 2. In this case, the pCO_2_ is within normal limits, and the primary disturbance appears to be a metabolic acidosis, as indicated by the low pH, low bicarbonate levels, and negative base excess.

**Table 2:** Arterial blood gas (ABG) findings. pH indicates the acidosis or alkalosis. Partial pressure of carbon dioxide (pCO_2_) reflects the effectiveness of ventilation and the respiratory component of acid-base balance. Partial pressure of oxygen (pCO_2_) assesses the efficiency of oxygen exchange in the lungs. Bicarbonate (HCO_3_⁻) represents the metabolic component of acid-base balance. Base excess indicates the amount of excess or insufficient base in the blood, aiding in the assessment of metabolic derangement.

Laboratory test	Result	Reference range
pH (arterial)	7.33	7.35-7.45
pCO_2_ (arterial)	39.0 mmHg	35-45 mmHg
pO_2_ (arterial)	151 mmHg	80-95 mmHg
HCO_3_ (arterial)	21.2 mmol/L	18-31 mmol/L
Base excess (arterial)	-4.9 mmol/L	-2-2.0 mmol/L

**Figure 1:**
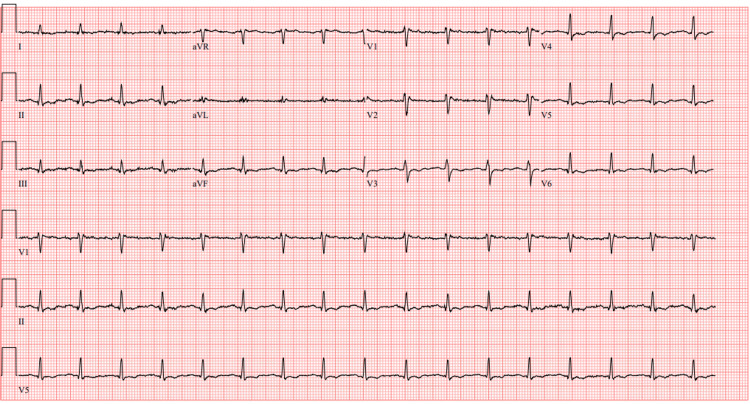
EKG showed sinus tachycardia, with nonsignificant T wave abnormality.

Electrocardiogram (EKG) showed sinus tachycardia, with nonsignificant T wave abnormality (Figure 1). EKG showed no ischemic changes, supporting the interpretation of elevated troponin as secondary to myocardial stress from cardiogenic shock.

**Figure 2:**
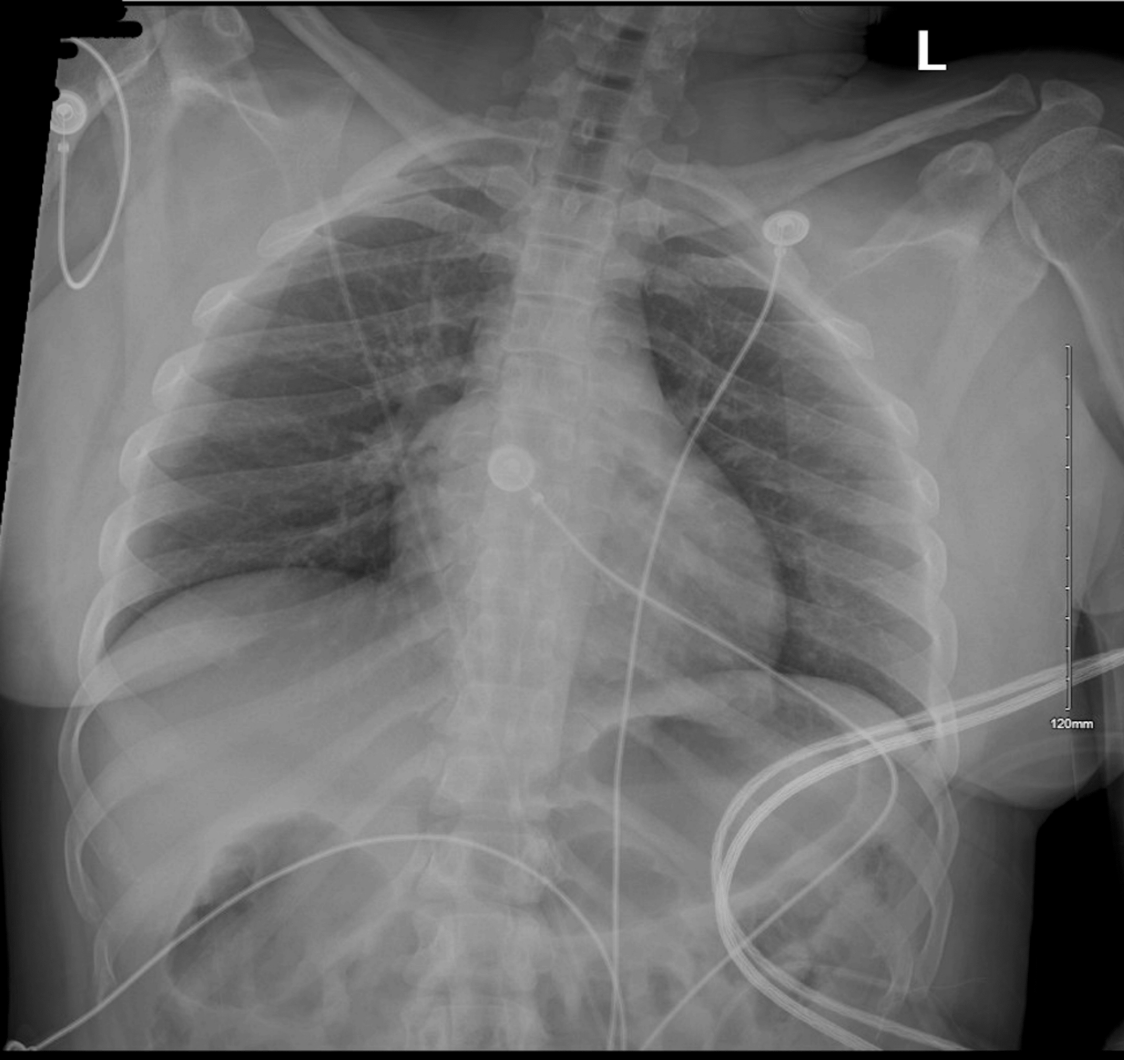
The chest X-ray was normal.

Chest X-ray was normal, ruling out acute pulmonary edema or infectious etiology in the presence of respiratory distress (Figure 2). The patient was initially treated with dextrose, glucagon, and calcium. Glucagon was administered to address severe hypoglycemia, and calcium was given to stabilize cardiac membranes and address potential hypocalcemia. IV hydrocortisone was administered in the ED, along with a 2-liter normal saline IV bolus, to manage lactic acidosis (lactate level 5.4 mmol/L).

The patient was admitted to the Intensive care unit (ICU) for cardiogenic shock secondary to suspected myxedema coma and was placed on pressor support along with inotropes. Initially started on IV pressor support (norepinephrine 8 mg in 250 mL D5W, dose titrated between 9.38 and 65.63 mL/h, vasopressin 40 units/100 mL infusion, IV dopamine at rate of 9.75 mL/h), IV hydrocortisone sodium succinate 100 mg every 8 hours, and IV levothyroxine 200 mcg reduced to 100 mcg daily. The initial high dose of levothyroxine (200 mcg) was chosen to rapidly correct the severe hypothyroid state, with subsequent reduction to 100 mcg daily to avoid over-replacement.

**Figure 3:**
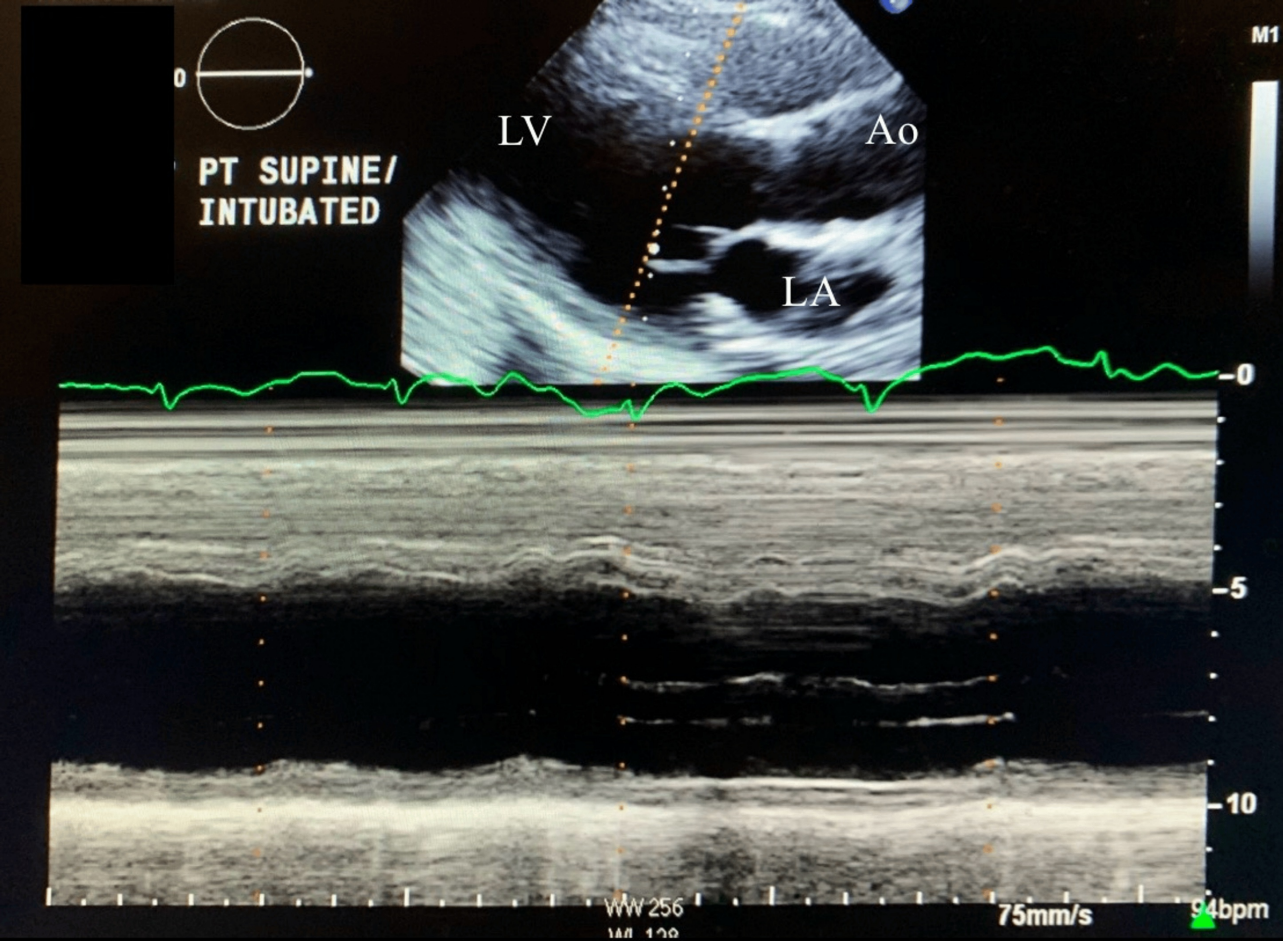
M mode echocardiography from parasternal long axis view showing ejection fraction of 10-15% on day one of admission. LV: left ventricle; LA: left atrium; Ao: aorta

Transthoracic echocardiogram (TTE) is notable for left ventricular ejection fraction (EF) of 10-15% with severely depressed left ventricular systolic function (Figure 3). There was akinesis of the mid to apical segments in the left ventricle as well. Initial TTE was consistent with cardiogenic shock. The patient was having difficulty protecting the airway, and there was a need for intubation.

Also, given the need for a higher level of care, the patient was transferred for further care to another healthcare institute, where the patient was sedated with fentanyl/propofol for the Critical-Care Pain Observation Tool (CPOT) <3. She was maintained on a ventilator with spontaneous breathing trials for a week. On day two, the patient was moving extremities without purpose, following concerns for an episode of seizure-like activity, and the patient was placed on levetiracetam. Neurology was consulted, who recommended video electroencephalogram (vEEG), and magnetic resonance imaging (MRI) of the brain. Upon a negative video electroencephalogram (vEEG) report, levetiracetam was discontinued. Neurology recommended follow-up as an outpatient. The patient was gradually weaned off pressure support. Magnetic resonance imaging (MRI) of the brain came negative for acute hemorrhage, mass effect, or infarction.

**Figure 4:**
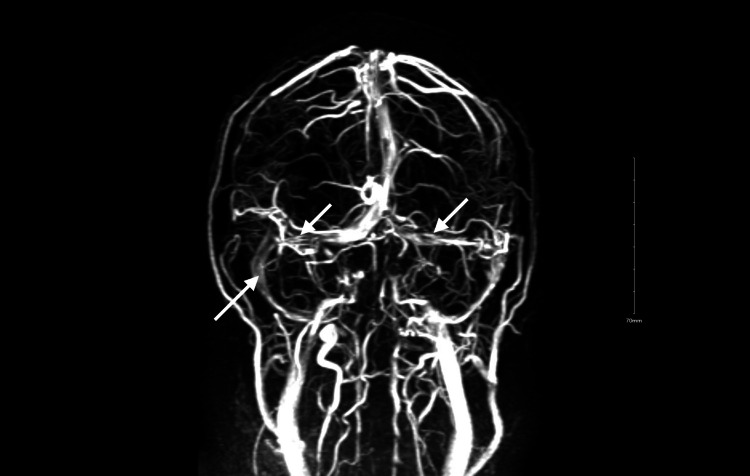
MRV brain with contrast showing bilateral stenotic transverse and sigmoid sinuses (white arrows). MRV: magnetic resonance venography

Magnetic resonance angiography (MRV) was suspicious for intracranial hypertension, bilateral stenotic transverse, and sigmoid sinuses (Figure 4). Neurosurgery was consulted and carefully reviewed the MRV, noting that the patient may have an alternate venous drainage pathway between vascular spaces communicating with the distal right sigmoid sinus. Conservative management was advised.

**Figure 5:**
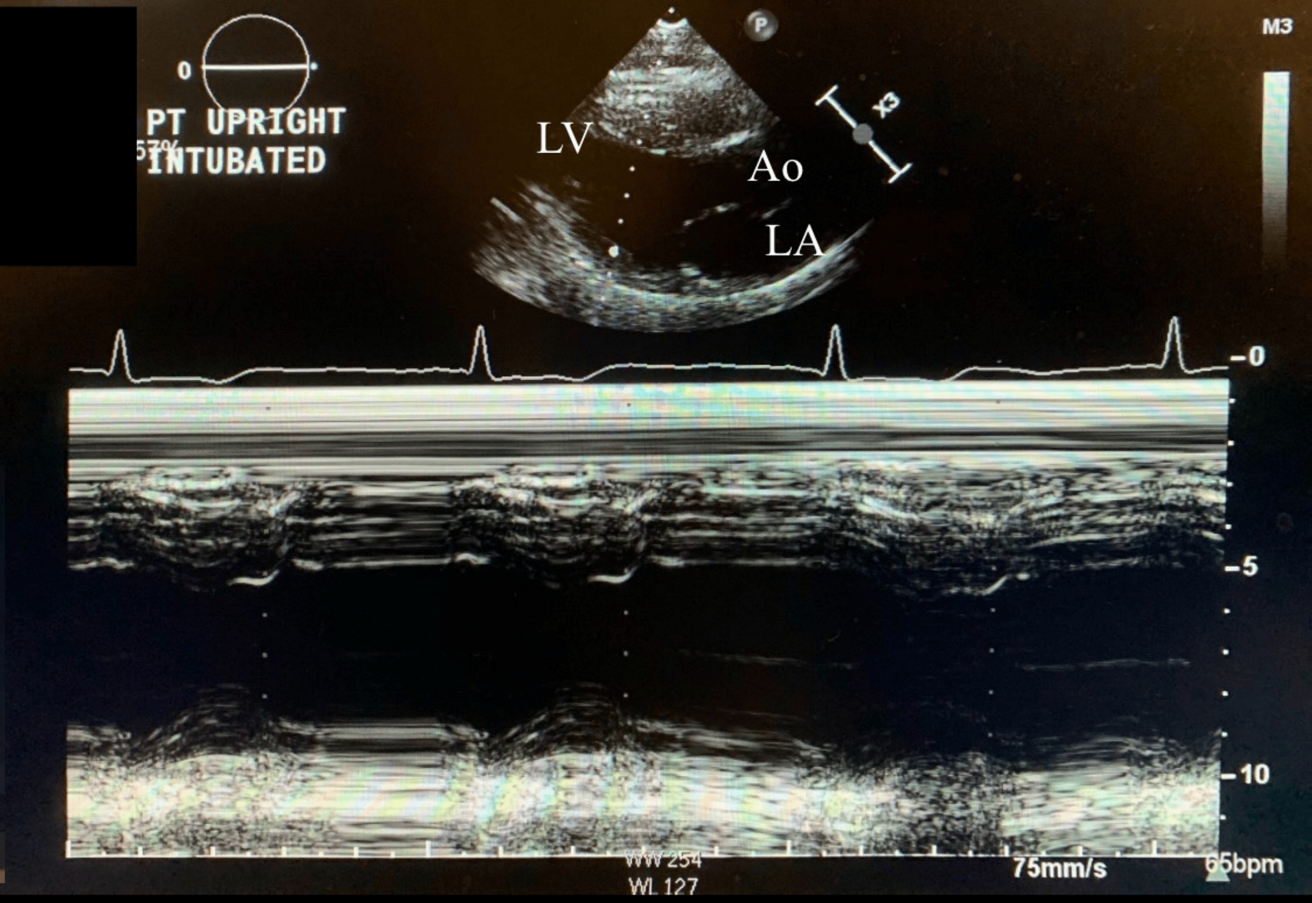
M mode echocardiography from parasternal long axis view showing ejection fraction of 50-60% on day seven of admission. LV: left ventricle; LA: left atrium; Ao: aorta

Serial TTEs suggested an improvement in EF from 5% to 60% gradually (Figure 5). Myocarditis workup came positive for coxsackie IgG 1:1600, but antiviral therapy was not initiated. The patient was extubated on day eight after successful spontaneous breathing trials, and the mental status returned to baseline, awake, alert, and oriented to time, place, and person. T4 returned to within normal limits. The patient was transferred back to our institute for further management of hypothyroidism. The patient's condition gradually improved with aggressive treatment, and she was discharged home following cardiac clearance with a home levothyroxine dose of 125 mcg daily along with hydrocortisone 10 mg twice a day (BID) as per endocrinology. The patient was advised to take the medications as prescribed and to follow-up with the primary care provider (PCP), cardiology, and endocrinology.

During follow-up, thyroid function tests demonstrated normalization of free T4 levels, and the patient reported improved energy levels and exercise tolerance. Her EF remained stable at 60% on repeat echocardiography, confirming sustained cardiac recovery. Endocrinology follow-up included adjustments in hydrocortisone dosing to ensure optimal adrenal function and comprehensive education on medication adherence and recognizing early signs of decompensation were key components of her discharge plan.

## Discussion

The management of myxedema coma requires immediate attention and aggressive treatment due to its high mortality rate, which can range from 30% to 60%, even with appropriate therapy [1,4]. This study highlights the critical importance of early intervention and multidisciplinary care in managing this critical endocrine emergency.

Pathophysiology

Thyroid hormone (TH) plays a crucial role in regulating cellular function throughout the body by regulating gene transcription. The primary active form, triiodothyronine (T3), is derived from thyroxine (T4) through deiodination [3]. TH influences multiple physiological processes such as an increase in Na-K-ATPase activity, carbohydrate metabolism, increase in free fatty acid (decreased cholesterol, phospholipids, and triglycerides), increased vitamin requirements due to enzyme induction, and overall acceleration of metabolism [3].

The most common causes of hypothyroidism in the United States and worldwide are autoimmune thyroiditis and iodine deficiency, respectively [5]. There are many precipitating factors, including but not limited to infection, trauma, hypoxemia, cerebrovascular accidents, congestive heart failure, amiodarone, lithium, and surgery [3].

In hypothyroidism, there is an alteration in renal function by a reduction in glomerular filtration rate (GFR) and sodium reabsorption, and impairment in free water excretion, all these mechanisms lead to hyponatremia, which is present in this case. Differential diagnoses are possible adrenal insufficiency or sepsis. 

Cardiogenic shock secondary to cardiac arrest in the presence of myxoedema coma

Myxedema coma profoundly affects the cardiovascular system, primarily due to the loss of thyroid hormone's regulatory effects on cardiac function. Decreased myocardial contractility and reduced cardiac output, compounding the effects of low cardiac output. This decreased perfusion to vital organs, including the brain and kidneys, exacerbated tissue hypoxia and metabolic acidosis. Persistent hypoperfusion and lactic acidosis led to a critical state of hemodynamic collapse and pulseless electrical activity (PEA) cardiac arrest [1-3]. Bradycardia is a characteristic feature resulting from both direct effects on the sinoatrial node and decreased sympathetic activity [2,5]. It can progress to various arrhythmias, including heart blocks and prolonged QT intervals [6,7].

While bradycardia is a feature of myxedema coma due to the reduced sympathetic response and diminished myocardial contractility associated with severe hypothyroidism, this patient presented to ED with tachycardia. Cardiogenic shock initiates a compensatory activation of the sympathetic nervous system, resulting in tachycardia. Also, the cardiac arrest and subsequent resuscitation efforts, including the administration of epinephrine, would acutely increase sympathetic tone, leading to an elevated heart rate upon return of spontaneous circulation, explaining tachycardia in this case.

Patients are observed to have initial diastolic hypertension due to increased peripheral vascular resistance, potentially progressing to hypotension in severe cases like myxedema coma [1]. It also increases the risk of development of pericardial effusion caused by increased capillary permeability and impaired lymphatic drainage [2,7,8]. The vascular system is affected through increased peripheral resistance and reduced blood volume, contributing to cold intolerance and increased risk of atherosclerosis [3,9]. These cardiovascular manifestations collectively increase the risk of life-threatening complications in severe cases. Understanding these pathophysiological changes is crucial for effective management and treatment of patients with myxedema.

Neurological manifestation of myxoedema coma

Typically, myxoedema coma presents as lethargy, which can be present for months and is followed by transient episodes of reduced consciousness, gradually progressing into a comatose stage. There are many other associated findings, such as depression, disorientation, decreased deep tendon reflexes, psychosis, slow mentation, paranoia, and poor recall [1]. The pathophysiology behind this is increased meningeal permeability and cerebral blood flow and a decrease in metabolism [3,10]. The neurological findings in this case, including the low GCS score and sluggish pupils, correlated with the severe metabolic derangements and hypoperfusion typical of myxedema coma. These neurological symptoms were corroborated by imaging findings, such as mild intracranial hypertension on MRV, ruling out acute ischemic or hemorrhagic changes.

Diagnosis

Myxedema coma remains primarily a clinical diagnosis, supported by laboratory findings of severe hypothyroidism (low free T4 and high TSH). However, in the acute setting, treatment should not be delayed while awaiting laboratory confirmation. Clinically, altered mental status and hypothermia are hallmark features, accompanied by signs consistent with hypothyroidism, including bradycardia, hypoventilation, hypotension, and hyponatremia. Laboratory findings for primary hypothyroidism reveal markedly low or undetectable levels of total T4, free T4, and free T3, with elevated or normal TSH. However, differential diagnoses must include secondary or tertiary hypothyroidism and euthyroid sick syndrome, where TSH elevation may be atypical.

In this case, TSH is suppressed along with low T3, and low T4, representing secondary hypothyroidism, secondary to prior pituitary tumor resection, complicating the recognition of hypothyroidism. Typical laboratory abnormalities in myxedema coma may be elevated creatine phosphokinase, which can be misled towards myocardial infarction [9]. Transaminases and lipids are often elevated due to metabolic dysregulation, while hypoglycemia and hyponatremia with low serum osmolality are also common [11].

A full septic workup is recommended to rule out infection, including blood cultures, lactate, and imaging. An electrocardiogram (EKG) may show bradycardia, low voltage, variable blocks, or prolonged QT interval. Arterial blood gas (ABG) analysis frequently indicates hypoxia, hypercapnia, and respiratory acidosis. Echocardiography may reveal reduced left ventricular function, pericardial effusion, or other cardiac abnormalities in cases complicated by cardiogenic shock [3]. Imaging studies showing cardiomegaly warrant further assessment for pericardial effusion. Neurological investigations, such as lumbar puncture, may reveal elevated protein, while electroencephalogram (EEG) changes are nonspecific [12].

A 2014 retrospective study suggested a diagnostic scoring system for myxedema coma, assessing 14 patients with myxedema coma and seven without. A score of ≥60 was indicative of myxedema coma, while scores between 45 and 59 signaled a heightened risk. This score evaluates dysfunction across thermoregulatory, central nervous, cardiovascular, gastrointestinal, and metabolic systems alongside the presence or absence of a precipitating event [13].

Management

The cornerstone of treatment for myxedema coma is thyroid hormone replacement. It is important to initiate intravenous levothyroxine (T4) at a loading dose of 200-400 mcg, followed by daily maintenance doses of 1.6 mcg/kg tailored to her weight to minimize risks of over-replacement and iatrogenic complications. T3 therapy was not administered due to the potential for arrhythmias, which can exacerbate cardiovascular instability, particularly in the setting of severe cardiogenic shock. While some experts advocate for the addition of T3 due to impaired peripheral conversion of T4 to T3 in severe hypothyroidism, recent literature suggests that T4 monotherapy may be sufficient in most cases [1,2,8].

Identifying and treating the precipitating factors is another crucial aspect of management [1,3]. Supportive care plays a vital role in our patient's management. This includes mechanical ventilation for respiratory support, fluid resuscitation to address hypotension while avoiding fluid overload, especially in patients with cardiogenic shock, and passive rewarming to manage hypothermia [3,8]. Stress-dose glucocorticoids (100 mg hydrocortisone every 8 h) are administered to address potential adrenal insufficiency, which is common in myxedema coma patients [3,14,15].

The pituitary-adrenal function may be impaired in cases with severe hypothyroidism. Restoring normal metabolic rates with exogenous thyroid hormones can risk precipitating adrenal insufficiency [3,15,16]. This particular patient has undergone pituitary tumor resection; therefore, administering glucocorticoids in stress doses is recommended to prevent this complication.

Cardiogenic shock requires inotropic support with dobutamine to improve cardiac output, and we closely monitored for arrhythmias, which are common in myxedema coma [8]. Echocardiography is ordered to assess left ventricular function and rule out pericardial effusion.

The patient's response to treatment is closely monitored through serial thyroid function tests, hemodynamic parameters, and neurological assessments. We collaborated with cardiology and endocrinology for management. Gradual improvement in mental status and hemodynamic stability is observed over the course of several days, consistent with the typically slow recovery seen in myxedema coma [3].

Long-term management focuses on optimizing thyroid hormone replacement by educating the patient on the importance of medication adherence to prevent recurrence and monitoring thyroid function by regular outpatient follow-up with cardiology and endocrine clinics [1,14,8].

This case underscores the complexity of managing myxedema coma and the need for a tailored approach based on individual patient factors. While our treatment strategy aligns with current guidelines, it's important to note that management protocols may vary between institutions, and ongoing research may lead to refinements in treatment approaches.

## Conclusions

Myxedema coma remains a challenging endocrine emergency with significant mortality risk, particularly when complicated by cardiogenic shock. Early recognition, prompt initiation of thyroid hormone replacement, and aggressive supportive care are crucial for improving outcomes. Further research is needed to optimize management strategies, perhaps having particular myxedema coma protocols in place to administer the medications promptly once recognized particularly in cases complicated by severe cardiovascular dysfunction.
